# IL-1β pre-stimulation enhances the therapeutic effects of endometrial regenerative cells on experimental colitis

**DOI:** 10.1186/s13287-021-02392-9

**Published:** 2021-06-05

**Authors:** Dingding Yu, Yiming Zhao, Hongda Wang, Dejun Kong, Wang Jin, Yonghao Hu, Yafei Qin, Baoren Zhang, Xiang Li, Jingpeng Hao, Guangming Li, Hao Wang

**Affiliations:** 1 Department of General Surgery, Tianjin Medical University General Hospital, 154 Anshan Road, Heping District, Tianjin, 300052 China; 2 Tianjin General Surgery Institute, Tianjin, China; 3 Department of Anorectal Surgery, the Second Hospital of Tianjin Medical University, Tianjin, China

**Keywords:** Endometrial regenerative cells, Colitis, Immunoregulation, Dickkopf-1, Mice

## Abstract

**Background:**

Ulcerative colitis (UC) is a chronic, relapsing, and non-specific inflammatory bowel disease, and the current treatment strategies were mainly used to relieve symptoms or for maintenance. Endometrial regenerative cells (ERCs) are mesenchymal-like stromal cells and have been demonstrated to alleviate multiple immune-dysregulation diseases. Pro-inflammatory stimuli were reported to enhance the immunosuppressive functions of ERCs, but the mechanism underlined is not fully understood. Here, we have designed this study to investigate the therapeutic effects of IL-1β-primed ERCs in the attenuation of experimental colitis.

**Methods:**

BALB/c mice were given 3% dextran sodium sulfate (DSS) for 7 consecutive days and free tap water for 3 days sequentially to induce experimental colitis. PBS (200 μL), ERCs, and IL-1β-primed ERCs (10ng/mL, 48 h) were injected (1 million/mouse/day, *i.v.*) on day 2, 5, and 8, respectively. Colonic and splenic samples were harvested on day 10 after DSS induction.

**Results:**

It was found that IL-1β-primed ERC treatment markedly attenuated colonic damage, body weight loss, and colon length shortening in colitis mice. Compared with other treatments, cell populations of CD4^+^IL-4^+^Th2 cells, CD4^+^CD25^+^FOXP3^+^ regulatory T cells (Tregs), and CD68^+^CD206^+^ macrophages in spleens were also significantly upregulated in the IL-1β-primed ERC-treated group (*p <* 0.05). In addition, lower expression of pro-inflammatory (IFN-γ, IL-17, TNF-α, and IL-6), but higher levels of anti-inflammatory cytokines (IL-4 and IL-10) were detected in colons in the IL-1β-primed ERC-treated group (*p <* 0.05 vs. other groups). Importantly, we also found that different generations of ERCs had an overall lower secretion of Dickkopf-1 (DKK1) by IL-1β pre-stimulation (*p <* 0.05) and a higher expression of β-catenin in colonic and splenic tissues after the administration of IL-1β-primed ERCs.

**Conclusions:**

This study has demonstrated that IL-1β pre-stimulation effectively downregulated DKK1 expression in ERCs, which in turn promoted the wnt/β-catenin pathway activation in colonic and splenic tissues. Consequently, IL-1β-primed ERCs exhibited an enhanced therapeutic effect in the attenuation of DSS-induced colitis.

## Introduction

Ulcerative colitis (UC) is a chronic, relapsing, and non-specific inflammatory disease of the intestine. In the last decades, the incidence and prevalence of UC have experienced a dramatic upward trend in Europe and America [1]. But, up to now, there are still no effective measures to control its development. Moreover, the consistent inflammation in colons may also increase the risk of suffering colorectal cancer [2].

Though the etiology of UC has not been defined clearly [3], accumulating evidences indicate that UC is correlated with the inappropriate immune response of mucosa toward luminal bacterial floral [4]. Till now, clinic treatments for UC mainly comprise four aspects: aminosalicylates, glucocorticoid (GC), immune-suppressants, and biologics. Disappointingly, these agents are mostly used to relieve symptoms or for maintenance and sometimes are not well tolerated [5]. Moreover, long-term administrations are always accompanied with various toxic effects [6, 7]. Therefore, seeking a novel therapy to supplement the existing treatment is in urgent need.

Mesenchymal stromal cells (MSCs) were primarily recorded as a group of non-hematopoietic, self-renewing, plastic-adherent, and fibroblast-like stromal cells [8]. Plenty of evidences have demonstrated that MSCs possess the immunomodulatory and anti-inflammatory specialties [9], assuming migrating to the injury sites to promote the tissue repair and modulating the function of immunocytes, such as T cells, B cells, dendritic cells (DCs), and macrophages [10–13]. Relying on these immunomodulatory properties, MSCs are exhibiting unique potentials in attenuating the development of UC. But, at the same time, the deficiencies of MSCs are gradually emerging, such as the invasive obtaining process, poor proliferation capacity, and less availability [14]. These restrictions sharply limit its application as a clinical therapy.

Endometrial regenerative cells (ERCs), a new type of mesenchymal-like stromal cells, were isolated from human menstrual blood and firstly demonstrated by Meng et al. in 2007 [15]. ERCs possess the similar phenotypic markers with MSCs (high expression of CD29, CD44, and CD90 molecules, and lack expression of CD45), but surmount the limits of traditional MSCs. Compared with MSCs, ERCs are with more outstanding advantages, including diverse differentiation potentials, immunomodulatory properties, non-invasive obtaining process, and high proliferative capacity without karyotypic abnormality [16]. We and others have previously reported the forcible therapeutic effects of ERCs on immune-related diseases such as acute liver injury, critical limb ischemia, renal ischemia reperfusion injury, pulmonary fibrosis, myocardial infarction, and so on [17–22]. Moreover, no serious immunological rejections were emerging against the human-derived ERCs when they were used to treat animal models [19]. Therefore, ERCs are being gained more and more attention in immune-disordered diseases, and their beneficial efficacy is being recognized by researchers.

Pro-inflammatory milieu within the site of inflammation was reported to alter the immunophenotype, differentiation capacity, and immunomodulatory functions of adult stem cells (ADSC) [23]. Exposure to IFN-γ, TNF-α, interleukin-1β (IL-1β), IL-6, and IL-23 could increase the immunosuppressive activity of ADSC [23, 24]. IL-1β is closely associated with UC activity and significantly enhanced to a higher level in UC patients at the acute phase. IL-1 receptor type 1 (IL-1R1), required for IL-1β signal transduction, is highly expressed in ADSC [25]. And priming ADSC with IL-1β can induce a reduction in the secretion of inflammatory mediators in LPS-activated microglial cells [26]. Additionally, the cell-free supernatant harvested from IL-1β-primed ADSC significantly inhibited T-cell activation in vitro [27].

Therefore, the present study was designed to study whether IL-1β pre-stimulation could enhance the therapeutic effects of ERCs on experimental colitis, and try to explain the potential mechanism, which may provide insight into novel strategies to enhance ERC immunoregulatory potency.

## Methods

### Animals

Male adult BALB/c mice, aged 8–10 weeks and weighing 20–24g (Aoyide Co., Tianjin, China), were caged in a comfortable experimental condition in the Animal Care Facility, Tianjin General Surgery Institute (Tianjin, China). Mice were provided with 1 week to adapt to the new surroundings and free access to ample tap water and mouse food constantly. Total experiments were all fulfilled based on the protocols approved by the Animal Care and Use Committee of Tianjin Medical University (Tianjin, China), according to the Chinese Council on Animal Care guidelines.

### ERC preparation

The research protocol for human origin cells has been approved by the Medical Ethics Committee of Tianjin Medical University General Hospital (IRB2020-YX-128-01), and the informed consents of using ERCs for the study were obtained from volunteer participants. Human ERCs were isolated from woman menstrual blood of volunteer donors by a density gradient centrifugation method in accord with the previous study [28]. In brief, the mononuclear cells were firstly separated from menstrual blood and then suspended in the Dulbecco’s modified Eagle’s medium (DMEM) which was supplemented with 1% penicillin/streptomycin and 10% fetal bovine serum. Then, cells were seeded in 10-cm dishes and cultured in an incubator at 37°C with 5% CO_2_. ERCs would adhere to the bottom of dishes after overnight incubation and the culture medium was changed every 2 days to wash away the non-adhered cells. Two weeks later, when cells expanded to 80–90% area of dishes and displayed a spindle-shaped morphology, we split and passaged down ERCs as the rate of 1:3. Typical cell surface markers of ERCs were detected by a flow cytometry as the previous study described [15].

In vitro, we harvested the 3rd to 7th generation of ERCs, divided each generation of cells into 2 groups (ERC group and IL-1β-primed ERC group), and inoculated them at a concentration of 3.5 × 10^5^/mL with 2.5-mL culture media. After being cultured for 48 h, supernatants in each group were collected to implement ELISA analysis. The fifth generation of ERC supernatants and cell lysates, the candidate for the following in vivo treatments, were further collected and prepared for ELISA and RT-PCR test to analyze DKK1 expression changes.

### ERC differentiation potential

The basic differentiation potential of ERCs and IL-1β-primed ERCs was measured. Briefly, the third generation of ERCs and IL-1β-primed ERCs (3 × 10^5^ cells) were inoculated in a 6-well plate. When the confluence of cells reached more than 80%, the medium was changed with a pre-configured adipogenic medium, which was constructed of DMEM, 10% FBS, 10^−6^ mol/L dexamethasone, 10 μg/mL insulin, 0.5 mmol/L isobutyl methylxanthine, and 60 μmol/L indomethacin (Sigma-Aldrich, USA). After the culture for 2 weeks, cells were harvested and fixed with 4% paraformaldehyde for 30 min and washed three times with PBS. Oil Red was applied to stain cytoplasmic fat.

Additionally, the above third-generation cells were also inoculated with the osteogenic medium, which contains 10^−8^ mol/L dexamethasone, 10 mmol/L β-glycerol phosphoric acid, and 100 mmol/L ascorbic acid (Sigma-Aldrich). On the 10th day, cells were washed with PBS and fixed with 4% paraformaldehyde. Following which, alkaline phosphatase solution was applied to staining for 30 min to evaluate the osteogenic capacity. Purple staining cued the synthesis of alkaline phosphatases by osteoblasts.

### Experimental groups

The experimental colitis was induced by supplying the mice with 3% (wt/vol) DSS (MP Biochemicals, USA) tap water as previous studies described [14]. In the current study, 24 BALB/c mice were randomly allocated into 4 groups: normal group, untreated group, ERC-treated group, and IL-1β-primed ERC-treated group (*n* = 6 per group). All experimental groups were firstly supplied with 3% (wt/vol) DSS soluted water for 7 days and then replaced with the non-DSS tap water for the following 3 days. ERCs or primed ERCs (5th generation) were suspended in phosphate-buffered saline (PBS) and injected into experimental mice (1 × 10^6^ cells/mouse, *i.v.*) on day 2, 5, and 8 according to the protocols verified by us and others [17, 29]. The untreated group was also given the equal volume of PBS (200 μL) as the control.

Mouse body weights, general conditions, and fecal characters were monitored and kept into records daily, convenient for the Disease Activity Index (DAI) assessment and other statistical calculations. DAI is an indicator for disease activity which can comprehensively reflect the progression of colitis in mice. Its score was calculated by assessing weight loss, fecal character, and stool blood, accord to the scoring system (Min = 0, Max = 4) reported by Murthy et al. [30].

On day 10, mice were sacrificed after being fasted for 8 h. Colons were dissected carefully from the ileocecal junction verge to the anus, and their lengths were measured. Then, samples were washed with PBS to clean away the contents and longitudinally severed into two parts. One part was fixed in 10% formalin buffer preparing for pathology analysis, and the other was reserved at −80 °C for other experiments. Spleen samples were also harvested and split into two parts. One was immediately ground in PBS for FACS; the other was stored at −80°C for the ELISA test.

### Pathological examination

Colonic samples fixed in formaldehyde, after undergoing the processes of dehydration and paraffin embedding, were sectioned on an ultra-microtome (LEICA, Germany) at a thickness of 5μm for hematoxylin and eosin (H&E) staining. Histopathological scores were evaluated and calculated in a double-blinded manner, based on the following criteria [31]: (a) inflammation severity 0 (physiologic inflammation), 1 (mild inflammation or prominent lymphoid aggregates), 2 (moderate inflammation), 3 (moderate inflammation associated with crypt loss), and 4 (severe inflammation with crypt loss and ulceration) and (b) crypt damage 0 (no destruction), 1 (1–33% of crypts destroyed), 2 (34–66% of crypts destroyed), and 3 (67– 100% of crypts destroyed). The two respective scores, inflammation severity and crypt damage, were summed together to drive the histopathological scores for evaluating colonic inflammation (maximum score 7).

### Immunohistochemistry staining

Colonic tissues fixed in formalin were embedded in paraffin, sliced into 5-μm-thick sections, and deparaffinized in xylene. To repair colonic antigens, sections were further microwaved in citrate buffer (pH 6.0) and then quenched endogenous peroxidase with 3% hydrogen peroxide in methanol for 30 min. Then, the sections were blocked with 3.0% bovine serum albumin (BSA) for 15 min and incubated with iNOS and CD206 primary antibodies at 4°C overnight (iNOS antibody, 1:200 dilution, Proteintech, USA; CD206 antibody, 1:100 dilution, Boster, China). On the other day, the sections were washed with PBS (pH 7.4) three times before incubating with anti-rabbit IgG antibody (1:200 dilution, Servicebio, China). After washing with PBS again, the sections were incubated with DAB substrate and counterstained with hematoxylin. After dehydration with a series of ethanol, the sections were mounted with neutral gum. The images of immunohistochemistry (IHC) were taken by a Leica microscope. And the ImageJ software (National Institutes of Health, Bethesda, MD, USA) was applied to analyze the number of macrophages.

### Flow cytometry analysis

Mouse spleens were respectively ground, filtered with sterilized meshes, and suspended in 2mL precooled PBS. Then, we lysed these erythrocytes in splenic suspension with RBC Lysis Solution (1x) (Solarbio, Beijing, China), washed twice, and resuspended the splenocytes with PBS to a concentration of 1 × 10^7^/mL. Fluorescent monoclonal antibodies against mouse CD4, IFN-γ, IL-4, IL-17, CD25, FOXP3, CD68, CD206, CD11C, MHCII, and CD86 were applied to detect the populations of Th1 (CD4^+^IFN-γ^+^), Th2 (CD4^+^IL-4^+^), Th17 (CD4^+^IL-17^+^), Treg (CD4^+^CD25^+^FOXP3^+^), macrophage (CD68^+^CD206^+^), and DC (CD11c^+^MHCII^+^/CD86^+^) cells by a FACS Canto II flow cytometer (BD Biosciences, America), as previously described [32]. In addition, to accurately identify the subpopulation of Th1, Th2, and Th17 CD4^+^ T cells, splenocytes were firstly incubated with a cell stimulation cocktail (including phorbol-12-myristate-13-acetate (PMA), ionomycin, brefeldin A, and monensin) (ebioscience Inc., San Diego, CA, USA) for 5 h before being stained with fluorescent antibodies. The statistics of various immunocyte proportions were analyzed by Flowjo X software.

### Enzyme-linked immunosorbent assay (ELISA)

ERC supernatants and cell lysates were collected and prepared for measuring DKK1 expressions. Besides, equal weight (30mg) of same area intestinal tissues in each group was gathered and grounded with high efficiency tissue lysate buffer (RIPA) and phenylmethyl sulfonylfluoride (PMSF) (Solarbio, Beijing, China) for testing the level of IFN-γ, IL-17, TNF-α, IL-6, IL-4, IL-10, and β-catenin. Briefly, ELISA was carried out according to the manufacturer’s instructions (Boster, Wuhan, China). The reaction absorbance was determined at 450 nm with the Microplate Reader (Tecan, Mannedorf, Switzerland) and each sample was performed in duplicates to lessen the error.

### Western blot

β-catenin expressions in the spleen and colon were further evaluated by western blot. Briefly, tissue proteins were extracted from different groups respectively and separated by 10% sodium dodecyl sulfate–polyacrylamide gel electrophoresis (SDS-PAGE) gels, followed by transferring to a PVDF membrane. Membranes were blocked with 5% skim milk in Tris-Tween-buffered saline (TBST) and incubated overnight at 4°C with primary anti-β-actin and anti-β-catenin antibodies (Abcam). After being washed in TBST for 3 times, each membrane was incubated with the secondary antibody at room temperature for 30 min. Anti-β-actin was used as the loading control. At last, the objective proteins were developed by an enhanced chemiluminescence detection system (BIO-RAD, USA).

### Real-time polymerase chain reaction (RT-PCR)

To further determine the transcriptional changes of inflammatory mediators in colons, colonic total RNA was extracted with an RNAprep Pure Tissue Kit (DP431, Tiangen Biotech Co. Ltd., Beijing). The pureness and concentration of RNA were determined with an UV spectrophotometer (SANYO, Japan) at the spectrum of 260 and 280nm. cDNA was generated from the obtained RNA by using a Fastquant RT kit (KR106, Tiangen Biotech Co. Ltd., Beijing). Real-time quantitative PCR (RT-PCR) was carried out by using SuperReal Color Premix kit (FP216, Tiangen Biotech Co. Ltd., Beijing), according to the recommended protocol. The primer sequences involved were designed as follows:

- Human GADPH: forward, 5′-ACAACTTTGGTATCGTGGAAGG-3′,

reverse, 5′-AAGTGGTCGTTGAGGGCAATG-3′;

- Human DKK1: forward, 5′-ATAGCACCTTGGATGGGTATTCC-3′,

reverse, 5′-CTGATGACCGGAGACAAACAG-3′;

- Mouse GAPDH: forward, 5′-AGGTCGGTGTGAACGGATTTG-3′,

reverse, 5′-TGTAGACCATGTAGTTGAGGTCA-3′;

- Mouse IFN-γ: forward, 5′-GCCGCGTCTTGGTTTTGCAG-3′,

reverse, 5′-TACCGTCCTTTTGCCAGTTCCTCCA-3′;

- Mouse IL-17: forward, 5′-TTTAACTCCCTTGGCGCAAAA-3′,

reverse, 5′-CTTTCCCTCCGCATTGACAC-3′;

- Mouse TNF-α: forward, 5′-CCCTCACACTCAGATCATCTTCT-3′,

reverse, 5′-GCTACGACGTGGGCTACAG-3′;

- Mouse IL-6: forward, 5′-TAGTCCTTCCTACCCCAATTTCC-3′,

reverse, 5′-TTGGTCCTTAGCCACTCCTTC-3′;

- Mouse IL-4: forward, 5′-ACAGGAGAAGGGACGCCAT-3′,

reverse, 5′-GAAGCCCTACAGACGAGCTCA-3′;

- Mouse IL-10: forward, 5′-AGAAGCATGGCCCAGAAATCA-3′,

reverse, 5′-GGCCTTGTAGACACCTTGGT-3′;

- Mouse β-catenin: forward, 5′-GAGTAGCTGCAGGGGTCCTC-3′,

reverse, 5′-GGACAGCAGCTGCGTATGTT-3′;

Each sample was performed in triplicates on MJ Research DNA Engine Opticon 2 PCR cycler (BIO-RAD, USA). The expressions of target genes among different groups were calculated with the comparative 2^−ΔΔCT^ method.

### Statistical analysis

Experimental data was presented as mean ± standard deviation (SD) and analyzed by SPSS 19.0. Data variance was evaluated by using one-way analysis of variance (ANOVA) (groups ≧ 3) or unpaired two-tailed student’s *t* test (groups = 2) after the normality test. The differences between groups were considered significant with *p* values ≤ 0.05 in statistics.

## Results

### ERC characters

**Fig. 1:**
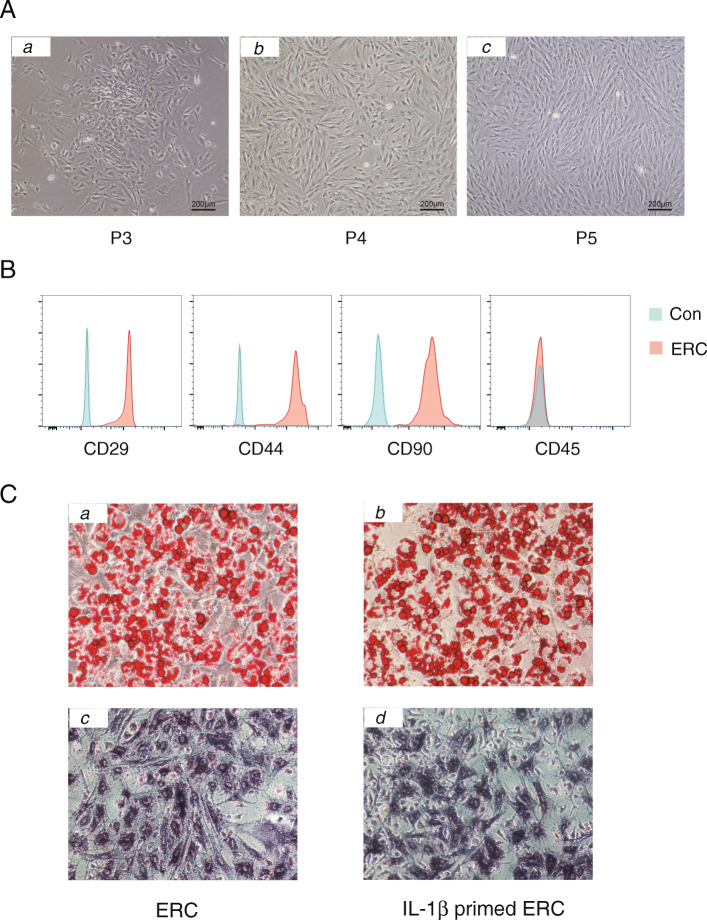
Morphology, phenotype, and differentiation potential of ERCs. **A** The 3rd–5th passage (P3–P5) ERC morphology. **B** Cell surface markers of ERCs were identified, indicating that ERCs highly expressed CD29, CD44, and CD90, but without expression of CD45. **C** Differentiation potential of ERCs and IL-β-primed ERCs. **a, b** Adipocytic differentiation. Red areas are lipid vacuoles which were stained by Oil Red. **c, d** Osteocytic differentiation. Purple staining indicated the synthesis of alkaline phosphatases by osteoblasts

We and others have previously demonstrated the differentiation potential and cytokine secretion profiles of ERCs [15], and the same methods were adopted for ERC extraction in the current study. In brief, the 3rd–5th passage (P3–P5) ERCs were collected and photographed. As shown in Fig. 1A, these cells presented a spindle-shaped morphology and displayed a high proliferation capacity. Additionally, cell surface markers of ERCs were identified (Fig. 1B), indicating that ERCs highly expressed CD29, CD44, and CD90, but did not express CD45. The basic differentiation potential of ERCs and IL-1β-primed ERCs was also measured. Specifically, abundant lipid vacuoles via Oil Red staining and alkaline phosphatase with purple staining were observed in cytoplasms of both ERCs and IL-1β-primed ERCs, indicating that both ERCs and IL-1β-primed ERCs could differentiate into adipocytes and osteoblasts (Fig. 1C).

### IL-1β-primed ERCs markedly ameliorated the symptoms of DSS-induced colitis

**Fig. 2:**
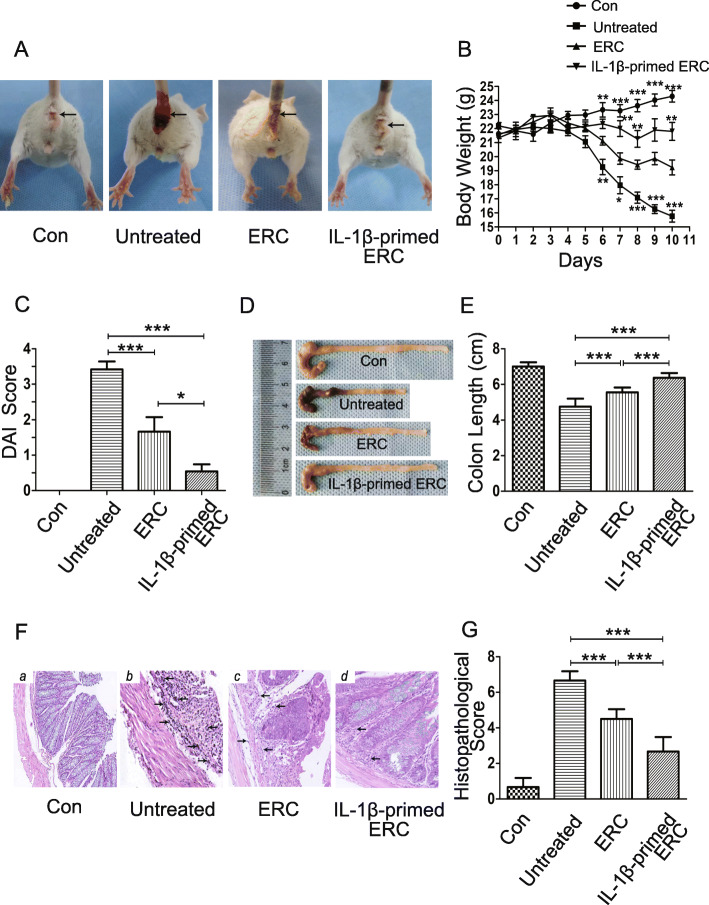
IL-1β-primed ERCs alleviate the symptoms of DSS-induced colitis. **A** Representative pictures showing bloody stool were taken on the 10th day after DSS induction. The mice in the IL-1β-primed ERC group were in the best condition than that in other groups. Body weight changes (**B**) and Disease Activity Index (DAI) score (**C**) of each group of mice were recorded daily. In the IL-1β-primed ERC group, the weight loss and DAI score were shown lesser than other groups. **D**, **E** The length of the colon in each group was measured and analyzed on the 10th day (*n* = 6). **F** Photograph (× 200, H&E staining) of representative histological sections of mouse colons in each group. Arrows indicated the inflammatory cell infiltration. **G** Histopathological scores were calculated according to the scoring system directed by Singh et al. [31] to assess the colonic injury quantitatively. Data were presented as mean ± standard deviation (SD) (*p < 0.05, **p < 0.01, ***p < 0.001). Statistical analysis was calculated by using one-way analysis of variance (ANOVA) followed by the least significant difference (LSD) test

In the present study, we used the 5th generation of ERCs for the following in vivo experiments. After 5 days of DSS induction, all the DSS-treated mice exhibited colitis with bloody stool, weight loss, and lethargy. But, following the treatment of ERCs, bloody stool (Fig. 2A) and body weight loss were found with moderate relieve and improvement (Fig. 2B, ERC group vs. untreated group, *p <* 0.001). Moreover, the therapeutic effects were further improved when the colitis mice were treated with IL-1β-primed ERCs (Fig. 2A, B, vs. ERC, *p <* 0.01).

In addition, we also analyzed the Disease Activity Index (DAI) score in each group. As shown in Fig. 2C, the DAI score was correspondingly decreased in the ERC group, when compared with that in the untreated group (*p <* 0.001). And in the IL-1β-primed ERC group, the DAI score was further decreased (*p <* 0.05, vs. ERC group). Given the above results, it suggests that IL-1β-primed ERCs have an enhanced therapeutic effect in alleviating the development of DSS-induced colitis.

### IL-1β-primed ERCs reduced histopathological damage of DSS-induced colitis

To observe the changes of colon morphology, colonic samples were collected and their lengths were measured (Fig. 2D, E). We found that the average length of colons in the untreated group is 4.8 cm (*n* = 6), indicating a significant reduction in colon length due to severe intestinal inflammation, while the average length of colons in the IL-1β-primed ERC group is 6.4 cm, higher than that of the ERC group (5.6 cm; Fig. 2E, *p <* 0.001). These results suggested that tissue injury and structural damage were obviously alleviated in the IL-1β-primed ERC group.

Pathological examination also confirmed the above findings. DSS intake caused severe injury, while in the IL-1β-primed ERC group (Fig. 2F (d)), the pathological condition of the colon was better, which showed slight damages to crypt structure, glands, and epithelium cells; mild inflammatory cell infiltration; and less goblet cell loss. Also, the histopathological score of the IL-1β-primed ERC group was lower than that of the ERC group (Fig. 2G, *p <* 0.001). Given together, these results indicate that IL-1β-primed ERCs could exhibit therapeutic effects in relieving histopathological damages in DSS-induced colitis.

### IL-1β-primed ERCs reduced Th1 and Th17, but enhanced Th2 cell and Treg populations in colitis mice

**Fig. 3:**
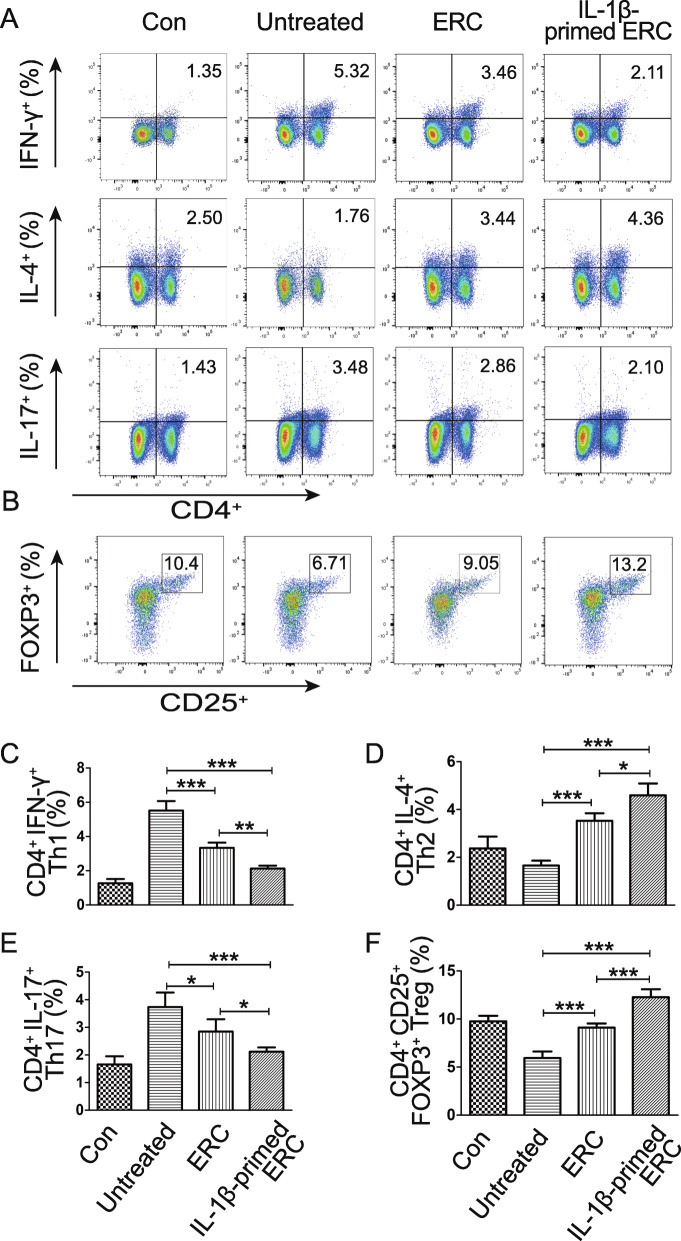
IL-1β-primed ERCs reduced Th1 and Th17, but enhance Th2 and Treg populations in colitis mice. Splenocytes were collected on the 10th day after DSS induction. To accurately identify the subpopulation of Th1, Th2, and Th17 cells, splenocytes were firstly incubated with a cell stimulation cocktail for 5 h before being stained with fluorescent antibodies. **A** Representative dot plots of CD4^+^IFN-γ^+^ Th1 cells, CD4^+^IL-4^+^ Th2 cells, and CD4^+^IL-17^+^ Th17 cells were shown while positive cells were counted from the quadrant Q2. **B** Dot plots of CD4^+^CD25^+^Foxp3^+^ Tregs. **C** Percentage of CD4^+^IFN-γ^+^ Th1 cells. **D** Percentage of CD4^+^IL-4^+^ Th2 cells. **E** Percentage of CD4^+^IL-17^+^Th17 cells. **F** Percentage of CD4^+^CD25^+^Foxp3^+^ Tregs. Data were mean ± SD (*n* = 6, *p < 0.05, **p < 0.01, ***p < 0.001). *p* values were calculated by one-way ANOVA followed by the LSD test

To determine the immunomodulatory effect changes in IL-1β-primed ERC, splenocytes from each group were prepared and stained for FACS analysis. As shown in Fig. 3A and B, we analyzed the proportion of CD4^+^IFN-γ^+^Th1, CD4^+^IL-4^+^Th2, CD4^+^IL17^+^Th17, and CD4^+^CD25^+^FoxP3^+^Treg cells, respectively. The statistical results in Fig. 3C–F show that the percentage of CD4^+^IFN-γ^+^Th1 and CD4^+^IL17^+^Th17 cells were significantly decreased in the ERC group when compared with that in the untreated group (Fig. 3C, E: Th1, *p <* 0.001; Th17, *p <* 0.05) and further decreased in the IL-1β-primed ERC group (ERC group vs. IL-1β-primed ERC group: Th1, *p <* 0.01; Th17, *p <* 0.05), whereas the proportion of CD4^+^IL-4^+^Th2 cells and CD4^+^CD25^+^FoxP3^+^ Tregs were increased in the IL-1β-primed ERC group (IL-1β-primed ERC group vs. ERC group: Th2 cells, *p <* 0.05; Tregs, *p <* 0.001). These results indicate that IL-1β pre-stimulation could augment the immunomodulatory function of ERCs, reflected in regulating Th1/Th2 paradigm, Th17, and Treg populations.

### IL-1β-primed ERCs reduced the population of mature DCs in colitis mice

**Fig. 4:**
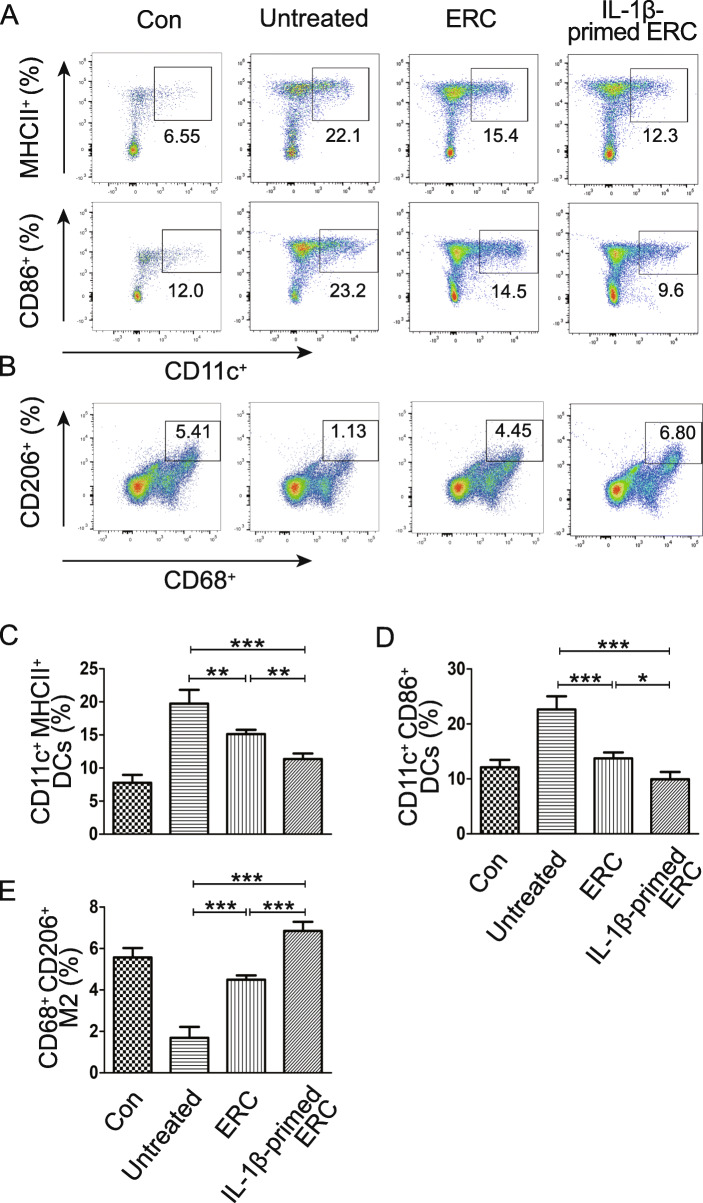
IL-1β-primed ERCs reduced the population of mature DCs, but increased M2 macrophages in colitis mice. To determine whether each treatment has an influence on regulating DC and macrophage phenotypes, anti-CD11c antibody and antigen presenting-related antibodies (anti-MHCII, anti-CD86) were used to measure mature DCs, while anti-CD68 antibody and anti-CD206 antibody were used for M2 phenotype macrophages in spleens. **A** Representative dot plots of CD11c^+^MHCII^+^ DCs and CD11c^+^CD86^+^ DCs in spleens. **B** Dot plots of CD68^+^CD206^+^ macrophages. **C**–**E** Percentage of CD11c^+^MHCII^+^ DCs, CD11c^+^CD86^+^ DCs, and CD68^+^CD206^+^ macrophages, respectively. Data were mean ± SD (*n* = 6, *p < 0.05, **p < 0.01, ***p < 0.001). *p* values were analyzed by one-way ANOVA followed by the LSD test

To explore the immunomodulatory effects of IL-1β-primed ERCs on the population of dendritic cells (DCs), mature DCs in splenocytes were detected with anti-CD11c and anti-MHCII/CD86 monoclonal antibodies. As indicated in Fig. 4, the two population of mature DCs (CD11c^+^MHCII^+^, CD11^+^CD86^+^) were both reduced in the ERC group (untreated group vs. ERC group: CD11c^+^MHCII^+^, *p <* 0.01; CD11^+^CD86^+^, *p <* 0.001). Moreover, these two populations of mature DCs were further strikingly reduced in the IL-1β-primed ERC group, when compared with those of the ERC group (CD11c^+^MHCII^+^, *p <* 0.01; CD11^+^CD86^+^, *p <* 0.05). These data indicate that IL-1β pre-stimulation could also enhance the capability of ERCs on inhibiting DC maturation.

### IL-1β-primed ERCs increased the population of CD206^+^ M2 macrophages in colitis mice

CD206^+^M2 phenotype macrophage is one of the main sub-types of macrophage which plays the anti-inflammatory role in the pathogenesis of experimental colitis. Our previous study has revealed that ERCs could promote the differentiation of macrophage to M2 sub-type. In the present study, to determine whether IL-1β pre-stimulation has an influence in regulating CD68^+^CD206^+^ M2 proportions, we measured splenocytes in different groups. Compared with that in the untreated group, the M2 population was obviously raised in the ERC group (Fig. 4B, E: untreated group vs. ERC group, *p <* 0.001). Moreover, the percentage of M2 cells was further increased to a higher level in the IL-1β-primed ERC group (IL-1β-primed ERC group vs. ERC group, *p <* 0.001). Taken together, these results demonstrate that IL-1β-primed ERCs have a more powerful role in promoting the increase of immunosupressive M2 phenotype cells, which would help diminish the injury from acute immune response in colons.

### IL-1β-primed ERCs modulated macrophage infiltration in colons

**Fig. 5:**
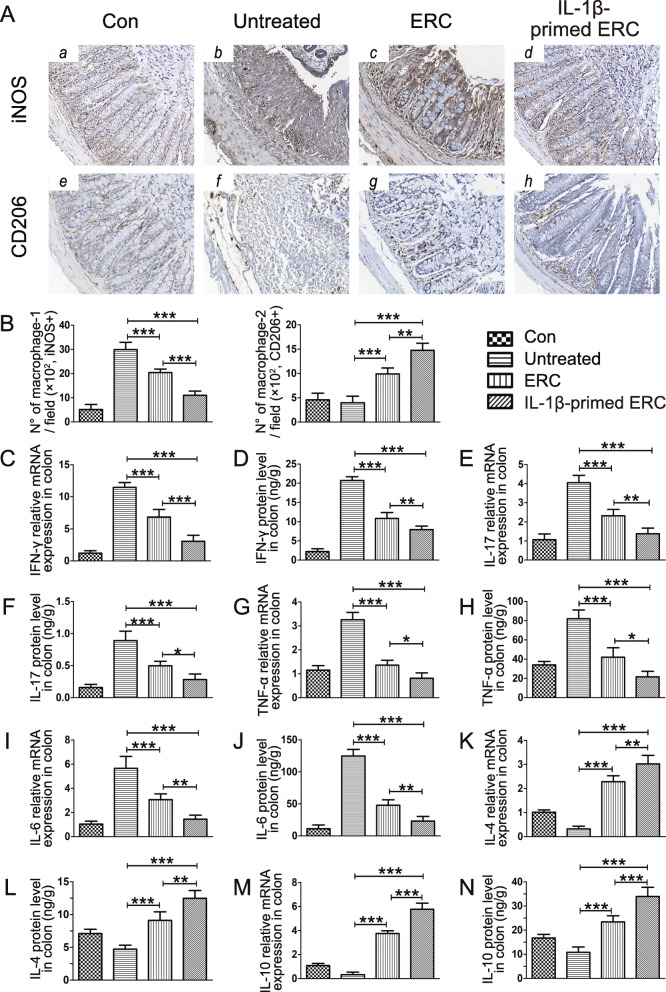
IL-1β-primed ERCs reduced macrophage infiltration and inflammatory cytokine expressions in colons. Intra-colon macrophage infiltration was evaluated by immunohistochemical staining. Specifically, we stained iNOS for detecting M1 cell infiltration, and CD206 for measuring M2 cell infiltration. The represent IHC images of mouse colons and quantitive data for cell counts of each group are shown in **A** (× 200) and **B**, respectively. The concentrations of inflammatory cytokine productions in colonic tissues were determined by ELISA kit and the relative mRNA expression changes were performed by real-time PCR. IFN-γ (**C**, **D**), IL-17 (**E**, **F**), TNF-α (**G**, **H**), IL-6 (**I**, **J**), IL-4 (**K**, **L**), and IL-10 (**M**, **N**) were shown, respectively, which are majorly secreted by Th1, Th2, Th17 cells, or CD206^+^ macrophages, and closely associated with the development of UC. Data were presented as mean ± standard deviation, and *p* values were calculated by using one-way ANOVA followed by the least significant difference (LSD) test (*n* = 6, *p < 0.05, **p < 0.01, ***p < 0.001)

Macrophage is centrally involved in the progress of colitis. In this study, we have already measured macrophage proportions in the spleen (Fig. 4B) and macrophage-related cytokine profiles in the colon (Fig. 5G–J). To further evaluate the modulatory role of IL-1β-primed ERCs on the local immune environment, we have performed the immunohistochemical staining to analyze the intra-colon macrophage infiltration in this colitis model. We stained iNOS for detecting M1 cell infiltration and CD206 for measuring M2 cell infiltration. As shown in Fig. 5A and B, the trend of macrophage infiltration in the colon (locally) was in accordance with that in the spleen (systemically, Fig. 4B). Specifically, iNOS expression tended to be decreased in the ERC-treated group when compared with that of the untreated group (*p* < 0.001). Moreover, as compared with the ERC-treated group, iNOS expression was further reduced in the IL-1β-primed ERC-treated group (*p* < 0.001). Conversely, when compared with the untreated group, CD206 expression was significantly increased in the ERC-treated group (*p* < 0.001), and further elevated in the IL-1β-primed ERC-treated group (*p* < 0.01). Given together, it indicated that treatment of IL-1β-primed ERCs effectively reduced intra-colon M1 cell infiltration, but increased M2 cell infiltration in this colitis model.

### IL-1β-primed ERCs altered the inflammatory cytokine expression profiles in colons

Inflammatory mediator expression disorders are demonstrated with the principle pathogenesis of colitis. In an attempt to address the immune modulation role of IL-1β-primed ERCs, we measured different inflammatory cytokine productions in colons, which are majorly secreted by Th1, Th2, Th17 cells, and CD206^+^ macrophages, and closely associated with the development of UC. As shown in Fig. 5C–J, the results demonstrated that IFN-γ, IL-17, TNF-α, and IL-6 in colons, both the mRNA and protein expression, were significantly reduced in the ERC group (vs*.* untreated group, Fig. 5C–J, *p <* 0.001) and further decreased in the IL-1β-primed ERC group (vs. ERC group, Fig. 5C, *p <* 0.01; Fig. 5D, *p <* 0.01; Fig. 5E, *p <* 0.01; Fig. 5F, *p <* 0.05; Fig. 5G, *p <* 0.05; Fig. 5H, *p <* 0.05; Fig. 5I, *p <* 0.01; Fig. 5J, *p <* 0.01).

In addition, the IL-4 and IL-10 levels in colonic tissues were apparently increased in the ERC group (vs. untreated group: Fig. 5K–N, *p <* 0.001). And in the IL-1β-primed ERC group, both of them were further increased (vs. ERC group: Fig. 5K, *p <* 0.01; Fig. 5L, *p <* 0.01; Fig. 5M, *p <* 0.001; Fig. 5N, *p <* 0.001). Taken together, these data indicate that IL-1β pre-stimulation could strikingly polish up the therapeutic effects of ERCs on altering inflammatory mediator expression profiles. And these effects are in line with the immunocyte proportion changes (Th1, Th2, Th17 cells, and CD206^+^ macrophages), which is mainly relying on promoting anti-inflammatory factor expression and inhibiting pro-inflammatory production.

### Pretreatment with IL-1β changed DKK1 expression in ERCs

DKK1 is a kind of secreted glycoprotein and expressed in mesenchymal stromal cells. Previous studies revealed that DKK1 played a role in promoting the development and differentiation of immunocytes which actively participates in the progression of inflammatory bowel disease.

Therefore, we designed to measure DKK1 secretion level in P3-P7 ERC culture supernatant. Interestingly, we found there is an overall lower secretion level of DKK1 in IL-1β-primed ERC supernatant, when compared with that in ERC culture supernatant (Fig. 1C). In addition, the 5th generation of ERCs were found with the lowest DKK1 secretion when compared with the other generations (*p < 0.05, **p < 0.01, ***p < 0.001).

**Fig. 6:**
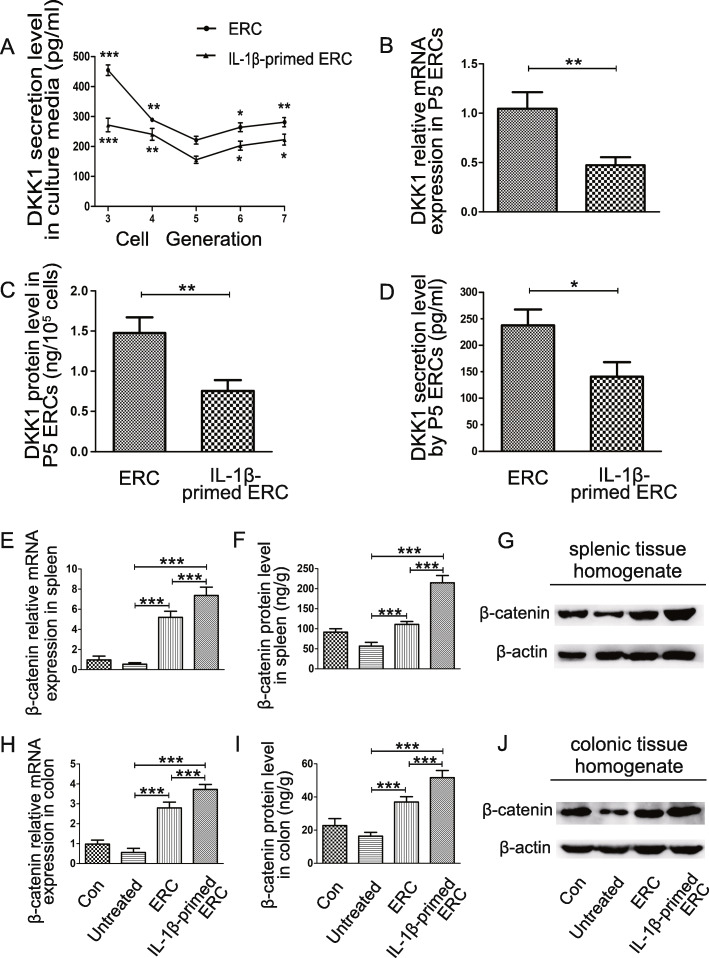
IL-1β-primed ERCs decreased DKK1 secretion, but promoted β-catenin expression in splenic and colonic tissues. **A** The DKK1 level in supernatants of 3rd–7th-generation ERCs were detected by ELISA kit. The 5th generation of ERCs were recorded with the lowest DKK1 expression when compared with other generations (vs*.* the 5th generation of ERCs, *p < 0.05, **p < 0.01, ***p < 0.001). **B** DKK1 mRNA expression in P5 ERCs (*n* = 3). **C** DKK1 protein expression in P5 ERCs (*n* = 3). **D** DKK1 secretion in P5 ERC supernatants (*n* = 3). **E**–**J** The mRNA expression level of β-catenin in spleens (**E**) and colons (**H**) was detected, respectively. Furthermore, the protein level of β-catenin in spleens (**F**) and colons (**I**) was also demonstrated. Western blot analysis of β-catenin protein expression in the spleen (**G)** and colon (**J**) tissue homogenate. Data were presented as mean ± SD (*p < 0.05, **p < 0.01, ***p < 0.001). Statistical analysis was performed by one-way ANOVA followed by the LSD test

Specifically, 5th-generation (e.g., P5) ERCs were collected to further clarify the DKK1 expression difference. As shown in Fig. 6B and C, DKK1 mRNA and protein expressions were decreased in IL-1β-primed ERC lysates (ERC vs. IL-1β-primed ERC: *p <* 0.01, Fig. 6B; *p <* 0.01, Fig. 6C). In addition, DKK1 secretion in ERC culture supernatant was also measured, which was shown in line with the trend found in ERC lysate expression changes (Fig. 6D). Overall, these results indicate that the 5th-generation IL-1β-primed ERCs would be the best source for application.

### IL-1β-primed ERC infusion increased β-catenin expression in the colon and spleen

In depth, DKK1 can uniquely inhibit the Wnt/β-catenin signaling transduction which exhibits anti-inflammatory effects. To determine whether IL-1β-primed ERCs have an influence on the activation of Wnt/β-catenin signaling, we measured the β-catenin expressions in colons and spleens, which is essential for the signaling transduction. As shown in Fig. 6, the β-catenin expression in colons and spleens, both at the mRNA and protein level, were increased in the ERC group (vs*.*untreated group, *p <* 0.001, Fig. 6E; *p <* 0.001, Fig. 6F; *p <* 0.001, Fig. 6H; *p <* 0.001, Fig. 6I). Moreover, in the IL-1β-primed ERC group, the β-catenin level was further significantly raised (vs. ERC group, *p <* 0.001, Fig. 6E; *p <* 0.001, Fig. 6F; *p <* 0.001, Fig. 6H; *p <* 0.001, Fig. 6I). Western blot analysis further confirmed the above findings (Fig. 6G, J). Taken together, these results suggested that IL-1β-primed ERCs exhibited anti-inflammatory effects in colitis through reducing DKK1 sections, which in turn leads to the activation of Wnt/β-catenin signaling.

## Discussion

UC is an inflammatory bowel disease characterized by diffuse inflammation of the colonic mucosa. Breakdown of immune homeostasis and imbalance of microenvironment are the main causes of recurrent colitis. Pro-inflammatory milieu within the site of inflammation was reported to alter the immunophenotype, differentiation capacity, and immunomodulatory functions of adult stem cells [23]. Of which, IL-1β is closely associated with UC activity and IL-1β-primed stem cells were reported toward an anti-inflammatory phenotype [25, 33]. Here, in the present study, we found that IL-1β-primed ERC treatment was also with an enhanced therapeutic effect, through ameliorating colitis symptoms, alleviating the pathological damages, and modulating the balance of immunocytes.

Specifically, ERCs were extracted based on our previously described protocols [15]. The harvested cells presented a spindle-shaped morphology and highly expressed CD29, CD44, and CD90, but did not express CD45, indicating that the extracted cells were with high purity. Additionally, in line with our previous findings [15], we also identified that ERCs and IL-1β-primed ERCs were capable of differentiating into adipocytic and osteogenic lineage cells in vitro, suggesting that IL-1β stimulation did not alter ERC differentiation potentials.

Experimental colitis was induced in BALB/c mice with the appearance of bloody stool, weight loss, and lethargy. Following the treatment with ERCs, the above symptoms were shown with moderate relieve and improvement. Moreover, the clinical characters were shown with strikingly ameliorated when administrated with IL-1β-primed ERCs. Pathological manifestations further confirmed the above findings. In the IL-1β-primed ERC group, the colonic average length was the highest, indicating slight intestinal inflammation. And the damages in glands, epithelium cell, and crypt structure were also slightest after the infusion with IL-1β-primed ERCs.

Additionally, our group has demonstrated that ERCs could survive and migrate to the damaged colon site in mice 24 h after ERC administration in this DSS-induced colitis model [16]. Similar results have also been reported by other researchers that human umbilical cord mesenchymal stem cells were found in the spleen, colon, and mesenteric lymph nodes on days 3 and 8 after cell injection [34]. These findings of cell tracking would support the notion that ERCs play immunomodulatory effects both locally and systemically.

As known, the balance between CD4^+^ T cells (Th1/Th2, Th17, and Tregs) is essential for sustaining the intestinal homeostasis and closely involved in the development of UC [35]. Markovic et al. have demonstrated that UC is a T-cell-driven disease which majorly attributes to the aberrant cytokine secretion by CD4^+^T cells [36]. Activated CD4^+^Th1 cells could produce pro-inflammatory cytokines (IFN-γ and TNF-α) and promote inflammation. In contrast, CD4^+^Th2 cells produce anti-inflammatory cytokines (IL-4 and IL-10) that suppress the inflammation progression in the gut. Besides, Li et al. [37] acclaimed Th17 cells also facilitate the induction of autoimmune tissue injury in colitis, while Tregs inhibit the damage. In our study, we analyzed the CD4^+^T cell population changes in splenocytes by flow cytometry and found that Th1 and Th17 populations in the IL-1β-primed ERC group were the lowest among experimental groups, while the populations of Th2 cells and Tregs in the IL-1β-primed ERC group presented the highest level. These data indicated that IL-1β priming could augment the immunomodulatory function of ERCs, at least in regulating Th1/Th2 paradigm, Th17 cell, and Treg populations.

Among immunocytes activated, DCs and macrophages are also involved in the development of DSS-induced colitis [38]. DCs, the major part of antigen-presenting cells (APCs), exert their effects by expressing costimulatory molecules (MHC class-II, CD86) on their cytomembranes [39] and could induce and promote T cell-mediated immune response in the site of injury. Macrophages are illustrated with a diverse plasticity, and their differentiation can be driven by the surrounding settings [40]. Macrophages generally differentiated into two phenotypic subsets: M1 pro-inflammatory sub-type and M2 anti-inflammatory sub-type [41]. M1 phenotype cells (data not shown), which are known as pro-inflammatory macrophages, have the ability to generate tumor necrosis factor alpha (TNF-α) and nitric oxide (NO) to exacerbate the inflammation [42], while M2 phenotype cells exhibit anti-inflammatory effects by producing IL-10, which mitigates the inflammation progress and sustains the intestinal immune homeostasis [43]. In the present study, we evaluated the population changes of macrophages and DCs in spleens. As the results showed, the population of DCs (CD11c^+^MHCII^+^ / CD11c^+^CD86^+^) in the IL-1β-primed ERC group was lower than that of other groups. Meanwhile, the population of M2 phenotype macrophages (CD68^+^CD206^+^) rise up to the highest level. Additionally, we have also analyzed intra-colon M1 and M2 sub-type macrophage infiltration. The results demonstrated that the intra-colon (locally) macrophage proportion changes were in accordance with that in the spleens (systemically), indicating that IL-1β-primed ERCs played a modulatory role in lessening pro-inflammatory M1 cell infiltration, and facilitating M2 cell infiltration. Taken together, these observations suggested that IL-1β-primed ERCs were with enhanced immunomodulatory effects on macrophages and DCs.

Accumulating documents showed that inflammatory mediators in colons (IFN-γ, IL-17, TNF-α, IL-6, IL-4, and IL-10) orchestrated the pathogenesis of UC temporally and specially [44, 45]. IL-4 and IL-10 are anti-inflammatory mediators with plenty of protective effects in colitis. IL-4 assists in inducing Th2 responses, inhibiting Th17 cell development, and polarizing macrophages toward M2 phenotype [46–48]. IL-10 participates in suppressing the antigen presentations and the synthesis of pro-inflammatory cytokines in colitis [49]. On the contrary, IFN-γ, IL-17, TNF-α, and IL-6 are pro-inflammatory factors usually strongly exacerbating the inflammation cascade in colitis [50, 51].

In this study, we have demonstrated that IL-1β-primed ERCs significantly enhanced the expressions of the anti-inflammatory mediators (IL-4 and IL-10), but decreased the levels of pro-inflammatory factors (IFN-γ, IL-17, TNF-α, and IL-6). Moreover, we found that the changes of these mediators were in line with the proportions of immunocytes, which further illustrate the effect of IL-1β-primed ERCs on immune homeostasis. Taken together, these results suggest that IL-1β priming could effectively improve the regulation of ERCs on inflammatory profiles during the pathogenesis of UC.

The encouraged therapeutic results in treating UC enlighten us the potential molecular mechanism in IL-1β-primed ERCs. DKK1 is a kind of secreted glycoprotein which can be secreted by MSCs [52]. DKK1 possesses a conservative gene sequence and previous studies revealed its expression in stromal cells can be downregulated by IL-1β. Relying on competing with Wnt ligands for LRP5/6 receptors [53, 54], DKK1 has a role in promoting the development and differentiation of immunocytes [55], such as macrophages, DCs, and CD4+ T cells, which actively participates in the progression of inflammatory bowel disease [56–58]. We have determined DKK1 levels in ERC and IL-1β-primed ERC culture supernatant. Our results showed that ERCs indeed secreted a considerable amount of DKK1, just like MSCs [59], and the DKK1 expression could be markedly downregulated after ERCs being stimulated by IL-1β at both the RNA and the protein levels.

**Fig. 7:**
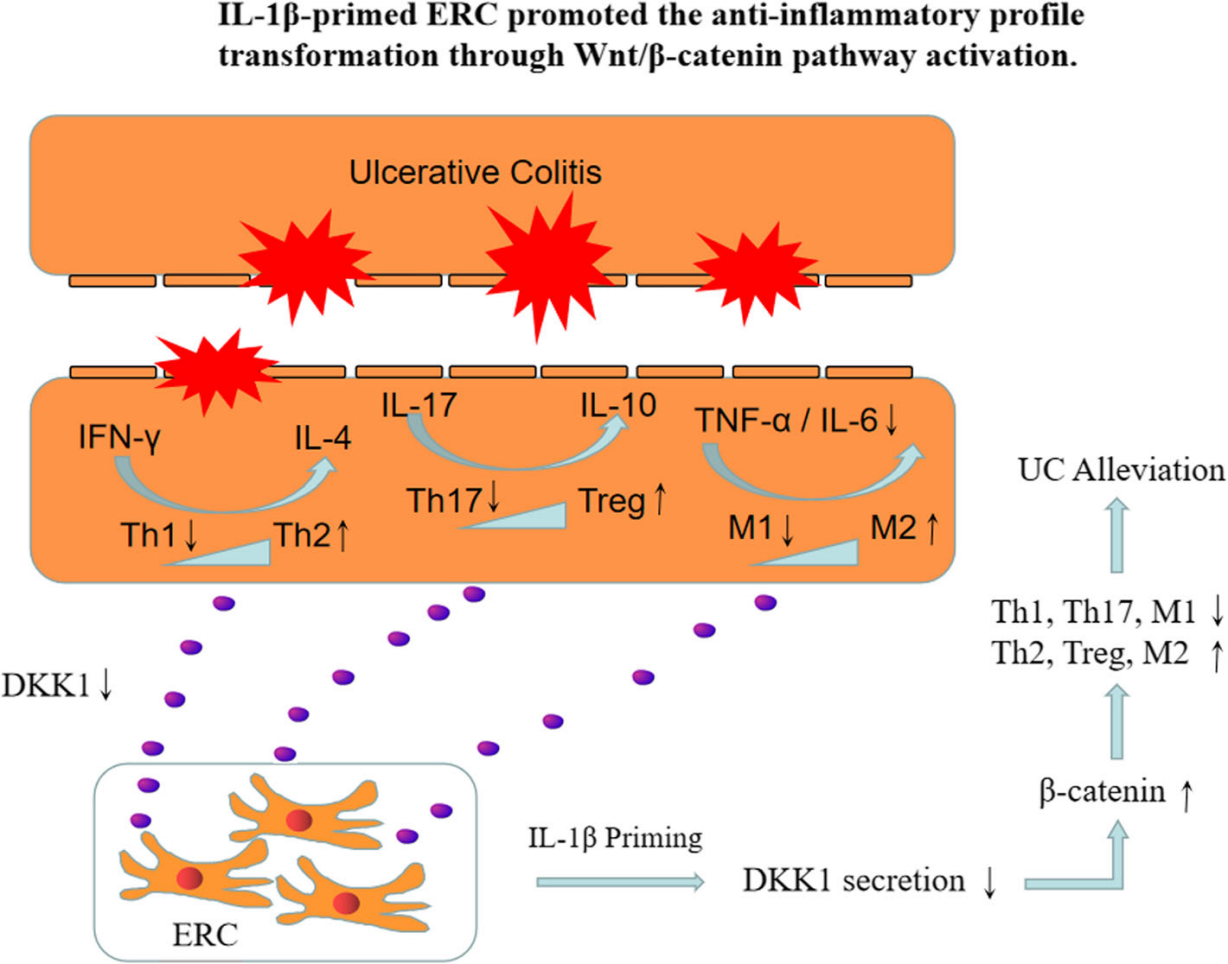
IL-1β-primed ERCs promoted the anti-inflammatory profile transformation through Wnt/β-catenin pathway activation. Wnt/β-catenin pathway is participating in regulating the development and differentiation of immunocytes and exhibiting anti-inflammatory effects in chronic disease. In the present study, DKK1 expression in ERC turned to be decreased after priming with IL-1β. After infusing IL-1β-primed ERCs into colitis mice, it was found that the β-catenin expression in splenic and colonic tissues were strikingly increased. Moreover, the inflammatory profile (Th1, Th17, M1, IFN-γ, IL-17, TNF-α, and IL-6) was transformed into an anti-inflammatory state (Th2, Treg, M2, IL-4, and IL-10), which is in line with the β-catenin expression changes. Therefore, in this study, we hypothesize that downregulating DKK1 expression in ERCs could promote the wnt/β-catenin pathway activation in immune cells, which would enhance the anti-inflammatory properties and optimize the immunomodulatory effect of ERCs

Wnt/β-catenin pathway is participating in regulating the development and differentiation of immunocytes [56] and exhibiting anti-inflammatory effects in chronic disease, while DKK1, relying on competing with Wnt ligands for LRP5/6 receptor, can uniquely inhibit the Wnt/β-catenin signaling transduction. And thus, DKK1 would block wnt/β-catenin activated anti-inflammatory effects [39]. Therefore, in this study, we hypothesize that downregulating DKK1 expression in ERCs could promote the wnt/β-catenin pathway activation, which in turn enhance the anti-inflammatory properties and optimize the immunomodulatory effect of ERCs (as shown in Fig. 7).

Thus, we have measured the β-catenin expressions, at both the RNA and the protein levels, to investigate the activity of canonical Wnt pathway. As expected, β-catenin production was significantly increased in the IL-1β-primed ERC group. Intriguingly, the high expression level of β-catenin was in accordance with the powerful immunoregulatory ability of IL-1β-primed ERCs in colitis. Thus, we concluded that reducing DKK1 expression in ERCs weakened the antagonistic effect of DKK1 on Wnt signaling, and then the Wnt/β-catenin pathway could be activated and β-catenin expression would be promoted, thereby improving the immunoregulatory and therapeutic effects of ERCs in the process of UC.

In this study, we have observed that the DKK1 reduction virtually improved the therapeutic effects of ERCs on UC. Moreover, we also found that the promising therapeutic effects of IL-1β-primed ERCs were achieved by activating the Wnt/β-catenin pathway, which would enrich the clinical therapeutic strategy on UC, and provide with a mechanism illustration on optimizing ERC-based cell therapy. However, the more detailed downstream gene expression changes concerning the Wnt/β-catenin pathway are still needed to be further explored.

In addition, we observed that ERCs downregulated DKK1 expression by IL-1β pre-stimulation, and IL-1β-primed ERCs attenuated the development of colitis. However, the whole-gene-wide sequencing on IL-1β-primed ERCs was warranted to evaluate the expression changes of other genes besides DKK1, whereas, in our study, we have provided a basic experimental evidence for enhancing the therapeutic effects of ERCs and offered a preliminary mechanism verification, which are promising findings and would facilitate, to an extent, ERC-based stem cell therapy application in clinic.

## Conclusions

Our present study has demonstrated that IL-1β-primed ERCs obviously exhibit a more effective immunoregulatory ability and better therapeutic effect in DSS-induced colitis. We also confirmed that IL-1β-primed ERCs could ameliorate the symptoms, alleviate the pathological damages, and modulate the balance of immunocytes in colitis mice. IL-1β-primed ERCs participated in modulating inflammatory factor profile deviations in colon tissues, thereby maintaining local immune homeostasis and reducing progressed damages. Taking together, these novel findings propose with an encouraging and viable method for facilitating ERC-based stem cell therapy on attenuation of UC.

## Data Availability

All data generated or analyzed during this study are included within the article.

## Abbreviations

DKK1: Dickkopf-1
ERCs: Endometrial regenerative cells
MSC: Mesenchymal stromal cell
PBS: Phosphate-buffered saline
GC: Glucocorticoid
IL: Interleukin
UC: Ulcerative colitis
IBD: Inflammatory bowel disease
DSS: Dextran sulfate sodium
DAI: Disease Activity Index
H&E: Hematoxylin and eosin
Th: Helper T cell
Tregs: Regulatory T cells
DCs: Dendritic cells
M2: Macrophage type 2 cells
APCs: Antigen-presenting cells
FACS: Fluorescence activating cell sorter
IFN: Interferon
MHCII: Major histocompatibility complex-II
ELISA: Enzyme-linked immunosorbent assay
RT-PCR: Polymerase chain reaction
GADPH: Glyceraldehyde 3-phosphate dehydrogenase
TNF: Tumor necrosis factor
COX-2: Cyclooxygenase-2
MPO: Myeloperoxidase
iNOS: Inducible nitric oxide synthase enzyme
ANOVA: Analysis of variance
LSD: Least significant difference
SD: Standard deviation
